# Radiation-Induced DNA Damage in Operators Performing Endovascular Aortic Repair

**DOI:** 10.1161/CIRCULATIONAHA.117.029550

**Published:** 2017-12-18

**Authors:** Tamer El-Sayed, Ashish S. Patel, Jun S. Cho, James A. Kelly, Francesca E. Ludwinski, Prakash Saha, Oliver T. Lyons, Alberto Smith, Bijan Modarai

**Affiliations:** 1Academic Department of Vascular Surgery, School of Cardiovascular Medicine and Sciences, King’s College London, BHF Centre of Excellence at Guy’s and St Thomas’ NHS Foundation Trust, London, United Kingdom.; Guy’s and St Thomas’ Cardiovascular Research Collaborative, From Guy’s and St Thomas’ NHS Foundation Trust, London, United Kingdom; Guy’s and St Thomas’ Cardiovascular Research Collaborative, From Guy’s and St Thomas’ NHS Foundation Trust, London, United Kingdom; Guy’s and St Thomas’ Cardiovascular Research Collaborative, From Guy’s and St Thomas’ NHS Foundation Trust, London, United Kingdom; Guy’s and St Thomas’ Cardiovascular Research Collaborative, From Guy’s and St Thomas’ NHS Foundation Trust, London, United Kingdom; Guy’s and St Thomas’ Cardiovascular Research Collaborative, From Guy’s and St Thomas’ NHS Foundation Trust, London, United Kingdom; Guy’s and St Thomas’ Cardiovascular Research Collaborative, From Guy’s and St Thomas’ NHS Foundation Trust, London, United Kingdom; Guy’s and St Thomas’ Cardiovascular Research Collaborative, From Guy’s and St Thomas’ NHS Foundation Trust, London, United Kingdom; Guy’s and St Thomas’ Cardiovascular Research Collaborative, From Guy’s and St Thomas’ NHS Foundation Trust, London, United Kingdom; Guy’s and St Thomas’ Cardiovascular Research Collaborative, From Guy’s and St Thomas’ NHS Foundation Trust, London, United Kingdom; Guy’s and St Thomas’ Cardiovascular Research Collaborative, From Guy’s and St Thomas’ NHS Foundation Trust, London, United Kingdom; Guy’s and St Thomas’ Cardiovascular Research Collaborative, From Guy’s and St Thomas’ NHS Foundation Trust, London, United Kingdom; Guy’s and St Thomas’ Cardiovascular Research Collaborative, From Guy’s and St Thomas’ NHS Foundation Trust, London, United Kingdom; Guy’s and St Thomas’ Cardiovascular Research Collaborative, From Guy’s and St Thomas’ NHS Foundation Trust, London, United Kingdom; Guy’s and St Thomas’ Cardiovascular Research Collaborative, From Guy’s and St Thomas’ NHS Foundation Trust, London, United Kingdom; Guy’s and St Thomas’ Cardiovascular Research Collaborative, From Guy’s and St Thomas’ NHS Foundation Trust, London, United Kingdom; Guy’s and St Thomas’ Cardiovascular Research Collaborative, From Guy’s and St Thomas’ NHS Foundation Trust, London, United Kingdom; Guy’s and St Thomas’ Cardiovascular Research Collaborative, From Guy’s and St Thomas’ NHS Foundation Trust, London, United Kingdom

**Keywords:** aortic aneurysm, DNA damage, endovascular, occupational exposure, radiation

## Abstract

**Background:**

Radiation exposure during fluoroscopically guided interventions such as endovascular aortic repair (EVAR) is a growing concern for operators. This study aimed to measure DNA damage/repair markers in operators perfoming EVAR.

**Methods:**

Expression of the DNA damage/repair marker, γ-H2AX and DNA damage response marker, phosphorylated ataxia telangiectasia mutated (pATM), were quantified in circulating lymphocytes in operators during the peri-operative period of endovascular (infrarenal, branched, and fenestrated) and open aortic repair using flow cytometry. These markers were separately measured in the same operators but this time wearing leg lead shielding in addition to upper body protection and compared with those operating with unprotected legs. Susceptibility to radiation damage was determined by irradiating operators’ blood in vitro.

**Results:**

γ-H2AX and pATM levels increased significantly in operators immediately after branched endovascular aortic repair/fenestrated endovascular aortic repair (*P*<0.0003 for both). Only pATM levels increased after infrarenal endovascular aortic repair (*P*<0.04). Expression of both markers fell to baseline in operators after 24 hours (*P*<0.003 for both). There was no change in γ-H2AX or pATM expression after open repair. Leg protection abrogated γ-H2AX and pATM response after branched endovascular aortic repair/fenestrated endovascular aortic repair. The expression of γ-H2AX varied significantly when operators’ blood was exposed to the same radiation dose in vitro (*P*<0.0001).

**Conclusions:**

This is the first study to detect an acute DNA damage response in operators performing fluoroscopically guided aortic procedures and highlights the protective effect of leg shielding. Defining the relationship between this response and cancer risk may better inform safe levels of chronic low-dose radiation exposure.

## Abstract 2

Supplemental Digital Content is available in the text.

**Editorial, see p 2417**

**Clinical Perspective**

**What Is New?**

- This is the first study to detect acute radiation-induced DNA damage in operators who carried out endovascular aortic repair by demonstrating an increase in the expression of DNA damage/repair markers, γ-H2AX, and phosphorylated ataxia telangiectasia mutated in their circulating lymphocytes immediately after procedures.
- In vitro irradiation studies demonstrated that operators had a variable susceptibility to radiation-induced DNA damage.
- The use of leg lead shielding abrogated the DNA damage response in operators.

**What Are the Clinical Implications?**

- Conventional dosimetry fails to account for the biological consequences of radiation exposure to operators during fluoroscopically guided procedures.
- Safe radiation exposure limits have been set without considering any interindividual differences in susceptibility to deleterious effects.
- Wearing lower leg protective lead shielding is essential for reducing scatter radiation-induced DNA damage.
- The use of cellular markers, including γ-H2AX and phosphorylated ataxia telangiectasia mutated, which readily lend themselves to high-throughput sampling, may facilitate individual risk profiling, improve our understanding of the mechanisms involved in occupational radiation-induced mutagenesis, and define optimal protection strategies.

Recent years have seen an exponential increase in the number of fluoroscopically guided cardiovascular interventions carried out by interventional cardiologists, electrophysiologists, and vascular surgeons, with many of these for pathologies previously treated by open surgery. Endovascular aortic repair (EVAR), for example, has become the mainstay of treatment in many institutions and is increasingly used for patients who would have been turned down for intervention 10 years ago.^1,2^ The growing number and complexity of procedures means that interventionists are exposed to higher amounts of radiation, a subject that is becoming increasingly topical.^3–7^ A recently published 15-year follow-up study of the EVAR trial, comparing endovascular and open aortic repair, reported an increased incidence of malignancy in patients treated by EVAR.^8^ There is, rightly so, a significant focus currently on reducing the patients’ exposure to radiation, but mounting evidence suggests that recurrent low-dose exposure to the practitioner is equally as important. Robust data collection to assess the risks posed to the interventionist is in its infancy, but a number of studies suggest a link to adverse health effects, including a higher risk of posterior subcapsular lens changes and malignancy.^9–11^ One recent study found a higher incidence of malignancy, including brain cancer, breast cancer, and melanoma, in interventionists who performed fluoroscopically guided procedures compared with those who had never performed these.^12^ A better understanding of the hazards of occupational radiation exposure requires sensitive tools to measure exposure at an individual level and clarification of the biological effects of exposure.

Circulating lymphocytes are particularly sensitive to radiation and may, therefore, offer the opportunity to study the acute biological consequences of low-dose exposure.^13,14^ Double-stranded DNA breaks induced by ionizing radiation lead to phosphorylation of the histone protein H2AX to form γ-H2AX, with the levels of γ-H2AX in the cell peaking half an hour after exposure to radiation.^15–17^ During the acute phase of exposure, DNA damage in lymphocytes also results in induction of a damage sensor known as the Mre11-Rad50-Nbs1 complex, which causes rapid phosphorylation of ataxia telangiectasia mutated (ATM) protein.^18–21^ This, in turn, leads to phosphorylation of downstream targets that act as cell cycle checkpoints, resulting in DNA damage-induced arrest at G1/S, S, and G2/M as part of the DNA repair process.^22–24^ Expression of phosphorylated ATM (pATM), a DNA damage response marker, and γ-H2AX, a marker of DNA repair, in circulating lymphocytes may, therefore, be a sensitive biomarker of radiation-induced DNA damage.^15–17,25,26^ The use of such biomarkers could facilitate a biological assessment of the effects related to radiation exposure. The current safe limits for low-dose occupational radiation exposure have been extrapolated from data obtained from individuals exposed to high doses (eg, atomic bomb survivors) and assume a linear, no-threshold relationship between exposure and cancer risk.^27^ Emerging data suggest, however, that there is variability in tissue response to radiation, the safe threshold may vary between individuals, and the risk relationship is not linear.^14,28^

The present study aimed to (1) study the biological effect of radiation exposure in operators by measuring pATM and γ-H2AX expression in circulating lymphocytes after EVAR, (2) examine individual operator sensitivity to radiation exposure using γ-H2AX levels as a biomarker, and (3) evaluate the protective effect of wearing lower leg lead shielding.

## Methods

### Study Participants

Blood samples were collected from vascular surgeons and interventional radiologists before, immediately after, and 24 hours after they performed endovascular and open aortic repairs. All operators had experience of EVAR procedures and beyond their learning curve. The branched EVAR (BEVAR)/fenestrated EVAR (FEVAR) procedures were performed by 1 of 4 operators, all of whom had experienced >100 of these procedures. Endovascular procedures consisted of standard infrarenal EVAR and complex thoracoabdominal, BEVAR, and FEVAR. This study was approved by the London–City & East Research Ethics Committee (16/LO/1111) following the principles of the Declaration of Helsinki, and written informed consent was obtained from each participant.

### Procedural Details

All EVAR procedures were carried out in a hybrid operating theater equipped with the Philips Allura Xper FD20 fixed X-ray imaging system (Philips Healthcare). Default settings used were a pulse rate of 7.5 pulses per second for background fluoroscopy and 2 frames per second for digital subtraction angiography acquisitions. For both fluoroscopy and cineangiography, an x-ray beam filtration of 1.5 mm Al combined with 0.4 mm Cu was used. The equipment setup and operating staff positioning were similar for IEVAR and BEVAR/FEVAR procedures and have been described previously.^7^ The fluoroscopy equipment was controlled by a senior radiographer for each procedure. At the start of each case, the under table lead shielding was specifically checked to ensure that it was in the optimal position. A ceiling-mounted lead shield was available and positioned at the operators’ discretion for each procedure. Mobile lead shields for the radiographer and anesthetist, lead garments (0.35 mm thickness), leaded thyroid collars, and leaded goggles were used for all endovascular cases. Leg lead shields were not routinely worn. A cohort of 6 operators (selected from the first cohort of 15 studied) were asked to wear lower leg lead shielding (0.5 mm thickness, XENOLITE–TB, DuPont Technology, Lite Tech, Inc) as additional protection to separately study the effect of radiation exposure on operators when legs were protected.

### Standard Dosimetry

Electronic dosimeters (Hitachi-Aloka Medical PDM-127; Hitachi Aloka Medical Ltd) were used to measure direct radiation exposure. These devices recorded cumulative measurements of the dose equivalence of absorbed radiation in micro Sieverts for each case. Dosimeters were attached to 3 different areas on the operator: (1) left breast pocket under the protective lead garment, (2) left breast pocket over the protective lead garment, and (3) left mid-leg. The dose-area product (DAP), fluoroscopy time, and air kerma dose were recorded for all procedures.

### Flow Cytometry

Venous blood samples were collected from operators in ethylenediaminetetraacetic acid tubes (BD Biosciences). Red blood cells were lysed using Pharmlyse (BD Biosciences) for 10 minutes and then washed in 0.5% BSA/PBS for 5 minutes at 4°C. Cells were fixed (Inside Fix, Miltenyi Biotec) for 10 minutes at room temperature, followed by staining with fluorescein isothiocyanate-conjugated mouse antihuman CD3 antibody (Miltenyi Biotec) for 30 minutes on ice in the dark. Cells were then permeabilized on ice (Permeabilisation Buffer A, Miltenyi Biotec) and washed twice before staining for γ-H2AX and pATM using antihuman allophycocyanin- and phycoerythrin-conjugated antibodies, respectively (BD Biosciences and BioLegend). Allophycocyanin- and phycoerythrin-conjugated IgG isotype control antibodies (BD Biosciences and BioLegend) were used in fluorescence minus one samples for appropriate gating of γ-H2AX and pATM, respectively. Samples were processed on a MACSQuant flow cytometer (Miltenyi Biotec) and analyzed using FlowJo software (FlowJo LLC). A more detailed analysis of γ-H2AX and pATM expression in subpopulations of CD3^+^ lymphocytes was also carried out (Methods in the online-only Data Supplement).

Samples from operators performing IEVAR were also analyzed for the expression of 8-oxoguanine DNA glycosylase-1,^29^ a dedicated DNA repair enzyme, with its expression directly correlating with DNA damage caused by base oxidation as opposed to double-stranded DNA breaks (see Methods and Table I in the online-only Data Supplement for a list of all antibodies used).

For each procedure, the opportunity was taken to collect blood samples from the patient to study γ-H2AX and pATM expression in their circulating CD3^+^ lymphocytes using the same methodology as outlined for operators above.

### Immunocytochemistry

Blood samples were collected, lysed, and fixed as described above. Samples were incubated with mouse antihuman CD3 immunomagnetic microbeads for 30 minutes, followed by positive selection of labeled lymphocytes using LS Columns (Miltenyi Biotec). Isolated CD3^+^ cells were permeabilized (0.5% Triton X-100 in PBS for 20 minutes), washed, and stained with mouse antihuman γ-H2AX (5µg/mL; BioLegend), followed by secondary staining with donkey Cy3-conjugated antimouse (5µg/mL; Jackson ImmunoResearch Laboratories). Cells were washed and mounted using DAPI gel mount (Sigma-Aldrich).

### Irradiation of Blood Samples In Vitro

Blood from 6 operators randomly selected from the entire cohort of 15 operators studied was collected in ethylenediaminetetraacetic acid tubes and exposed to radiation doses between 100 and 1000 mGy using a Darpac 2000 (Gulmay Medical) x-ray unit (energy: 80 kVp [half-value layer, 2.0 mm AL], 6.9 mA, applicator: 8 cm diameter) positioned ≈25 cm from the x-ray source. After red blood cell lysis, γ-H2AX and pATM staining and analysis by flow cytometry (as described above) was carried out within 30 minutes of irradiation. Blood was collected and irradiated on 3 separate occasions from each operator, ensuring that they had not performed any intervention involving exposure to radiation in the 48 hours before sampling.

### Statistical Analysis

Data were analyzed using GraphPad Prism 7.0a (GraphPad Software Inc) and SPSS-22 (SPSS Inc). Where appropriate, nonparametric Wilcoxon signed rank, Mann-Whitney U and 2-way analysis of variance tests were used. A *P* value <0.05 was considered to be statistically significant.

## Results

### Peri-Operative Changes in γ-H2AX and pATM

**Figure 1.**
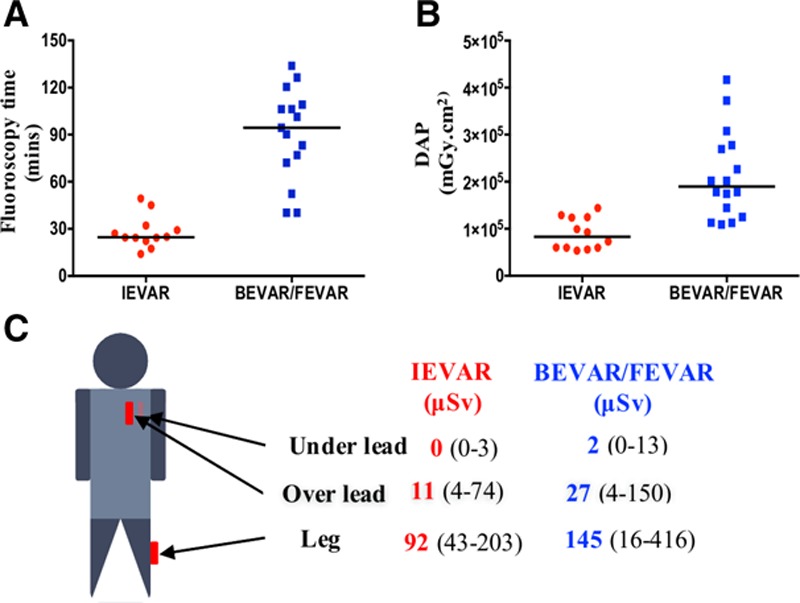
**Radiation exposure to operators during EVAR procedures.** **A**, Screening time during BEVAR/FEVAR was significantly longer than IEVAR (*P*<0.0001). **B**, DAP during BEVAR/FEVAR was higher than IEVAR (*P*<0.0001). **C**, Readings from personal dosimeters placed over operators’ chest under the lead, over the lead, and at the left leg. Leg doses were significantly higher during BEVAR/FEVAR compared with IEVAR (*P*<0.05). BEVAR indicates branched endovascular aortic repair; DAP, dose area product (mGy cm^2^); EVAR, endovascular aortic repair; FEVAR, fenestrated endovascular aortic repair; horizontal line, median; and IEVAR, infrarenal endovascular aortic repair.

**Figure 2.**
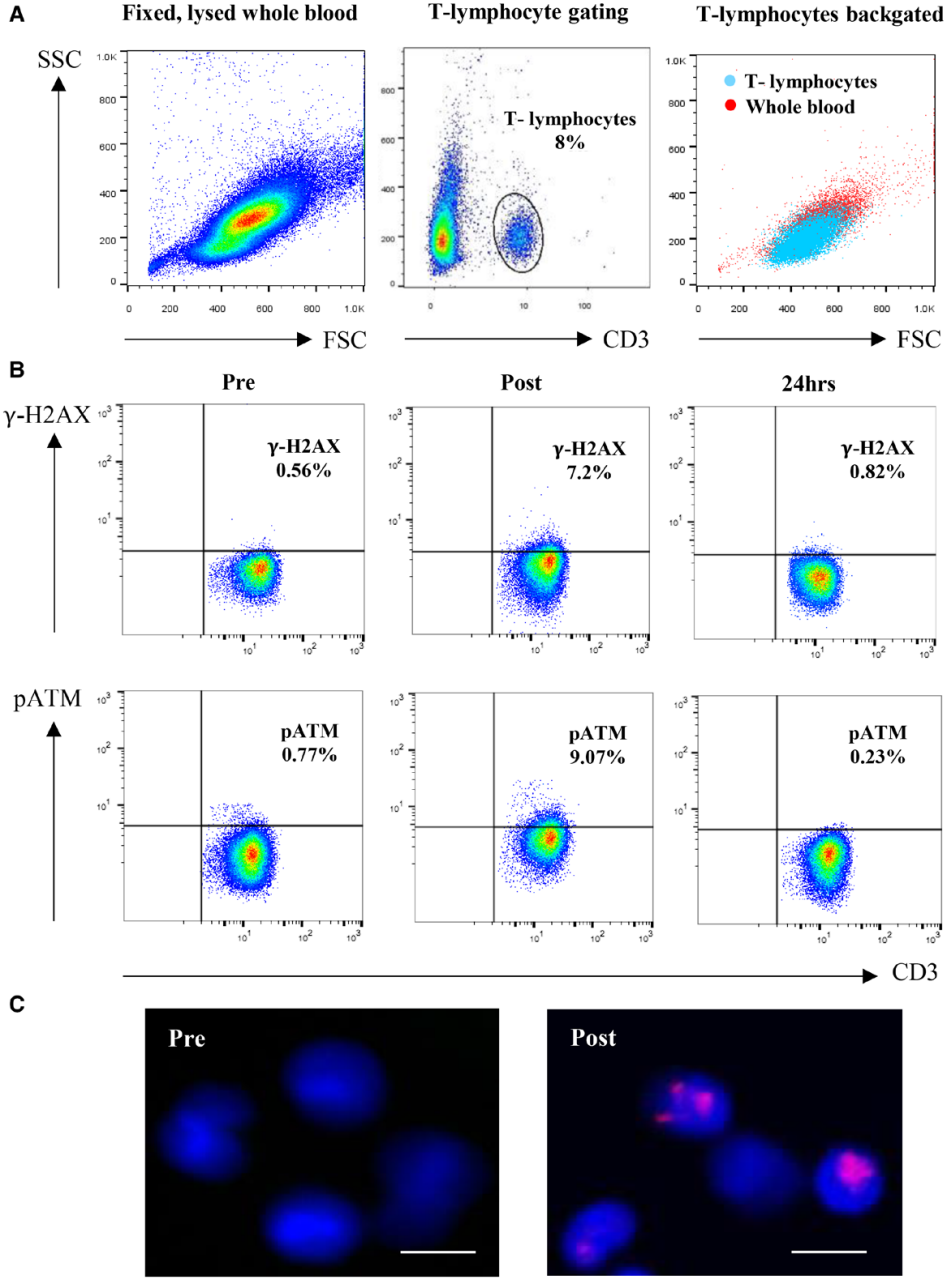
**Flow cytometric and immunohistochemistry analysis of γ-H2AX and pATM expression in operators’ lymphocytes during the peri-operative period of EVAR.** Flow cytometric dot plots of lysed, fixed, and permeabilized cells from whole blood collected from an operator before, immediately after, and 24 hours after FEVAR. **A**, Lymphocytes are identified according to forward and side scatter profile and gated according to the expression of CD3. **B**, Example flow cytometric dot plots showing that γ-H2AX expression in CD3^+^ lymphocytes increases in an operator immediately after a FEVAR and falls to preoperative levels after 24 hours. This response is also seen with lymphocyte expression of pATM. **C**, Immunohistochemical staining of lymphocytes (DAPI, blue) isolated from an operator shows, compared with the preoperative sample, an increase in γ-H2AX expression (purple foci) on these cells immediately after FEVAR. γ-H2AX indicates gamma H2AX; DAPI, 4’,6-diamidino-2-phenylindole dihydrochloride; EVAR, endovascular aortic repair; FEVAR, fenestrated endovascular aortic repair; and pATM, phosphorylated ataxia telangiectasia mutated protein (scale bar=10 µm).

Fifteen operators (13 males, 40 years of age [34–49]) (Table) carried out 45 procedures, including 15 IEVAR, 16 BEVAR/FEVAR, and 14 open abdominal aortic aneurysm repairs. BEVAR/FEVAR was associated with longer screening time and higher DAP (*P*<0.0001 for both) compared with IEVAR (Figure 1). Personal dosimetry showed minimal exposure under the operators’ protective lead garment but higher exposure over the lead (*P*<0.0001), particularly at the lower leg level (*P*<0.0001) (Figure 1). An optimized flow cytometric strategy was used to quantify both γ-H2AX and pATM expression in circulating lymphocytes (Figure 2). Immunohistochemistry confirmed that γ-H2AX foci, absent in both operator and patient blood samples preoperatively, appeared in postoperative lymphocyte films (Figure 2C).

**Table.**
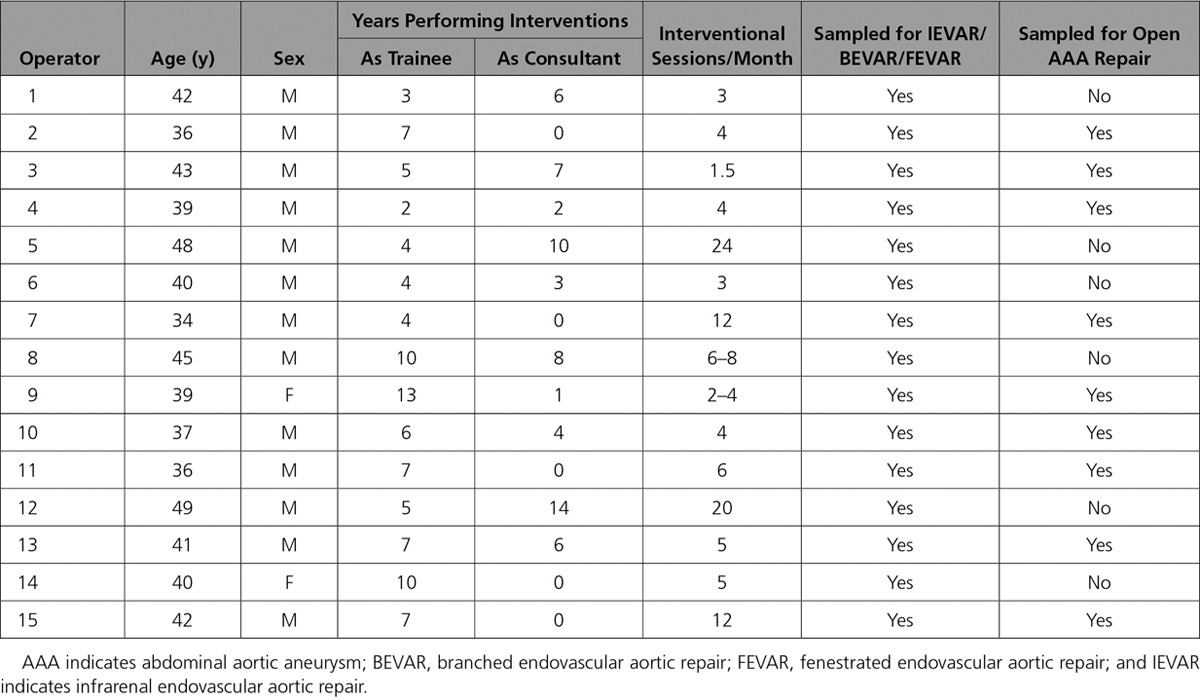
Details of Operators Participating in the Study

**Figure 3.**
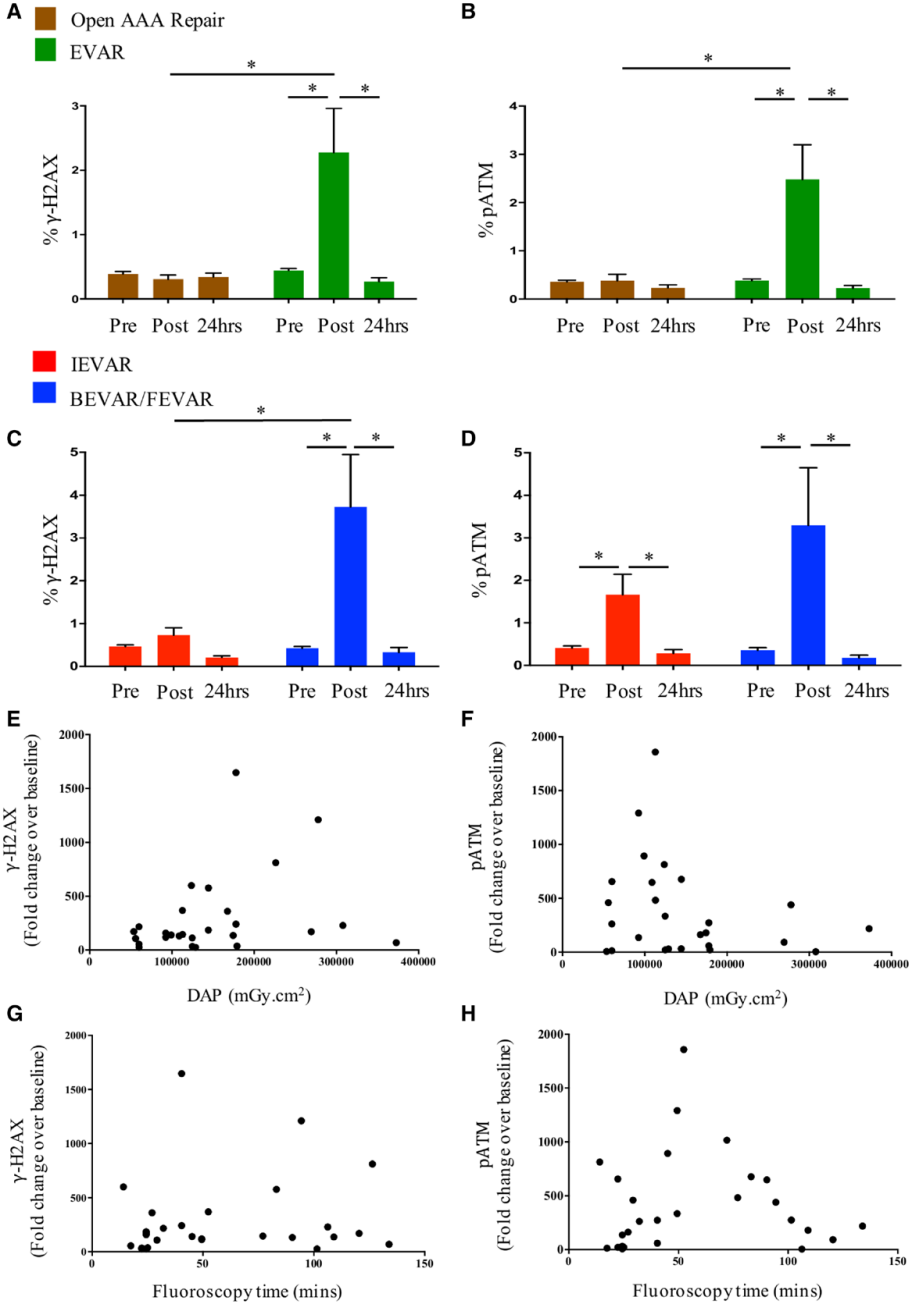
**Changes in expression of γ-H2AX and pATM in operators’ lymphocytes in response to radiation exposure during EVAR.** **A**, Expression of γ-H2AX in operators’ lymphocytes before, immediately after, and 24 hours after open aortic repair (n=14) and EVAR (all IEVAR and BEVAR/FEVAR procedures grouped together [n=31]). **B**, Expression of pATM in operators’ lymphocytes before, immediately after, and 24 hours after open aortic repair (n=14) and EVAR (n=31). **C**, γ-H2AX expression during the peri-operative period of BEVAR/FEVAR (n=16) compared with IEVAR (n=15). **D**, pATM expression during the peri-operative period of BEVAR/FEVAR (n=16) compared with IEVAR (n=15). **E**, Correlation between fold change increase in γ-H2AX expression and DAP (n=31). **F**, Correlation between fold change increase in pATM levels and DAP (n=31). **G**, Correlation between fold change increase in γ-H2AX levels and fluoroscopy time (n=31). **H**, Correlation between fold change increase in pATM levels and fluoroscopy time (n=31). None of the operators studied wore leg shielding during these procedures. γ-H2AX indicates gamma H2AX; BEVAR, branched endovascular aortic repair; EVAR, endovascular aortic repair; FEVAR, fenestrated endovascular aortic repair; IEVAR, infrarenal endovascular aortic repair; and pATM, phosphorylated ataxia telangiectasia mutated protein. *P<0.05.

A significant increase occurred in the levels of both γ-H2AX and pATM in circulating lymphocytes of operators immediately after BEVAR/FEVAR (*P*<0.0003 for both) (Figure 3). The expression of pATM increased in operators who carried out IEVAR (*P*<0.04), but γ-H2AX did not show significant changes in this cohort. The expression of both markers fell to baseline levels in all operators after 24 hours (*P*<0.003 for both) (Figure 3). There was no change in γ-H2AX or pATM expression at any time point during the peri-operative period of open abdominal aortic aneurysm repair.

A significant postoperative rise in γ-H2AX and pATM in both T helper and cytotoxic T cell subpopulations of CD3^+^ lymphocytes was detected after EVAR, with the relative expression of γ-H2AX higher in T helpers compared with cytotoxic T cells (*P*<0.05 for all) (Figures I and II in the online-only Data Supplement).

The deeper phenotyping strategy used to compare the relative postoperative levels of γ-H2AX and pATM in CD4^+^ and CD8^+^ T lymphocytes showed that the increases in γ-H2AX levels were significantly higher in CD4^+^ naïve and central memory cells (Figures I and II in the online-only Data Supplement).

After IEVAR, a significant increase in 8-oxoguanine DNA glycosylase-1 expression occurred in the CD3^+^ lymphocytes that expressed elevated levels of pATM but not γ-H2AX (*P*<0.03) (Figure III in the online-only Data Supplement).

Changes in γ-H2AX and pATM in operators after BEVAR/FEVAR and IEVAR did not correlate with either DAP or screening time in either cohort (Figure 3).

In patients, increased expression of both γ-H2AX and pATM levels were detected in CD3^+^ lymphocytes immediately after BEVAR/FEVAR and IEVAR procedures (*P*<0.004 for both) (Figure IV in the online-only Data Supplement).

### Factors Affecting γ-H2AX Expression in Operators

**Figure 4.**
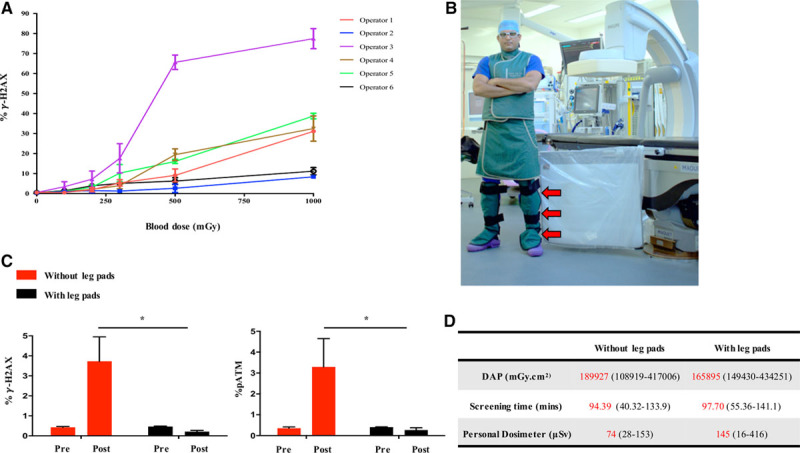
**Factors affecting γ-H2AX expression in operators.A**, Variation in γ-H2AX levels in operators’ lymphocytes after in vitro irradiation of their blood samples on 3 separate occasions (bars represent standard error of means, n=6, *P*<0.0001). **B**, An operator wearing lower leg shielding (red arrows). **C**, γ-H2AX and pATM expression in operators’ lymphocytes with (black bars, n=9) and without (red bars, n=16) the use of leg protection during BEVAR/FEVAR procedures (*P*<0.05). **D**, Radiation exposure measured by DAP and personal dosimeters worn at leg levels during procedures with (n=9) and without (n=16) leg shielding. γ-H2AX indicates gamma H2AX; BEVAR, branched endovascular aortic repair; DAP, dose area product; FEVAR, fenestrated endovascular aortic repair; pATM, phosphorylated ataxia telangiectasia mutated protein. *P<0.05.

A variable response (*P*<0.0001) was apparent when γ-H2AX expression in lymphocytes, sampled from 6 operators, was studied in vitro after controlled irradiation using doses ranging between 100 and 1000 mGy (Figure 4A). At any given dose, some operators mounted a consistently exaggerated response, whereas others demonstrated a far lower expression of γ-H2AX on their lymphocytes.

The same 6 operators were asked to wear lower leg shielding during BEVAR/FEVAR procedures (n=9 cases), and blood samples were obtained before and after they had performed each procedure. Wearing lower leg lead protection significantly abrogated the lymphocyte γ-H2AX and pATM response in operators after BEVAR/FEVAR, with no change in the expression of either marker immediately after the procedure (Figure 4C). Comparable DAP, over the leg exposure measurements and screening times, suggested that the radiation exposure associated with procedures during which the operators wore lower leg lead shielding was comparable with those carried out without leg protection (Figure 4D).

## Discussion

Ionizing radiation can induce different forms of DNA damage, such as base pair damage, single-stranded breaks, and double-stranded breaks, and the latter is considered the most deleterious because these are more difficult to repair than other forms of DNA damage.^30,31^ Left unrepaired, double-stranded DNA breaks can cause chromosomal instability and cell apoptosis. Incomplete repair leads to deletions and chromosomal rearrangements such as translocations and inversions that can ultimately lead to mutations.^20^ These types of chromosomal abnormalities have been detected in lymphocytes of both patients and hospital staff after chronic exposure to low-dose radiation.^13,16,17^

Circulating lymphocytes are particularly sensitive to radiation exposure, mounting an acute response to radiation-induced DNA damage, which includes raised expression of pATM and γ-H2AX. Elevated levels of the latter marker have been demonstrated in patients’ lymphocytes after pediatric cardiac catheterization and as a consequence of radiation exposure during diagnostic CT scanning.^17,32^ The present study demonstrates an upregulation of both γ-H2AX and pATM expression in interventionists’ and patients’ lymphocytes after endovascular aortic repair.

Neither γ-H2AX nor pATM has previously been studied in interventionists performing fluoroscopically guided aortic procedures. To our knowledge, the present study is the first to demonstrate an elevated expression of these markers of DNA damage/repair in operators exposed to radiation. This finding is of importance to the entire community of workers exposed to low-dose radiation. A more profound effect was seen in operators performing BEVAR and FEVAR, complex and lengthy repairs associated with higher radiation exposure compared with standard IEVAR. We and other groups have previously reported that DAP, fluoroscopy, and radiation exposure is higher for these complex procedures compared with standard IEVAR.^4,33^ In our experience, BEVAR/FEVAR was associated with a 2-fold increase in DAP and 3-fold longer fluoroscopy time.^5^ The present study confirmed, by personal dosimetery, an ≈2-fold higher exposure at leg level for the operator performing BEVAR/FEVAR as opposed to IEVAR.

Changes in γ-H2AX and pATM were not detected in operators after open repair, highlighting that this effect is directly related to radiation exposure. This effect was absent in operators who wore lower leg lead shielding during complex aneurysm repair, indicating that the majority of DNA damage occurs in lymphocytes irradiated in the lower leg tissues and long bones.

Our data also demonstrate interindividual variability in the induction of γ-H2AX in operators’ lymphocytes when irradiated in vitro, suggesting that susceptibility to DNA damage may vary and safe exposure limits may not apply universally.^32^ A range of doses, including some far higher than those recorded for occupational exposure, were used to provoke an exaggerated response and help unmask differences among individuals. Although an increased expression of both of these markers was detected immediately after endovascular intervention, levels fell back to normal 24 hours after the procedure in operators, a finding previously reported in patients exposed to radiation.^15–17^ A greater understanding of this reparative response to DNA damage is needed to determine the influence of factors such as age, sex, comorbidities, and chronicity of exposure and whether a complete repair is achieved in damaged cells. Estimates of cancer risk from exposure to ionizing radiation are based on epidemiological studies of exposed human populations, especially the atomic bomb survivors of Hiroshima and Nagasaki. These studies have provided relatively reliable estimates of risk for moderate to high radiation doses.^17,27,34,35^ However, risk estimates for repeated exposures to low-dose radiation are based on linear extrapolation using epidemiological data from high-dose exposures, making these estimates less reliable.^36–38^ Traditional methods to quantify radiation risk associated with fluoroscopically guided procedures is through absorbed radiation dose or exposure indices such as DAP, which estimate absorbed radiation dose. These methods provide a theoretical risk estimate that does not factor into individual variations in susceptibility to radiation damage. Measurement of biological markers, such as γ-H2AX and pATM, provides the opportunity for individual risk profiling, but at this stage a better understanding of the long-term consequences of the raised levels of γ-H2AX and pATM during endovascular interventions is required.

We found a postoperative rise in γ-H2AX and pATM in both T helper and cytotoxic cell populations, with the relative expression of postoperative γ-H2AX higher in T helper (CD4^+^) compared with cytotoxic T cells (CD8^+^). Using this deeper phenotyping strategy to compare relative expression levels in CD4^+^ and CD8^+^ cells, we found that postoperative γ-H2AX levels are significantly higher in CD4^+^ naïve and central memory cells. It appears that γ-H2AX did not rise in CD8^+^ central memory cells postoperatively.

Expanding on the biological significance of the differential expressions that we have found in T-cell subsets is beyond the scope of the present study. The effects of chronic radiation exposure on the overall health of surgical operators may only be revealed through long-term investigations. Other groups have found that CD8^+^ cells are more sensitive to radiation-induced apoptosis than CD4^+^ cells.^39^ It is possible that CD8^+^ cells are less likely to persist after being irradiated and be registered as γ-H2AX-expressing cells.

After IEVAR, there is a significant increase in 8-oxoguanine DNA glycosylase-1 expression, a dedicated DNA repair enzyme that specifically excises 7,8-dihydro-8-oxoguanine, with its expression directly correlating with DNA damage caused by base oxidation.^29^ Our finding of higher 8-oxoguanine DNA glycosylase-1 expression in CD3^+^ lymphocytes that demonstrate elevated levels of pATM but not γ-H2AX suggests that the DNA damage response marker, pATM, is activated in the lymphocytes of operators in response to DNA damage caused by base oxidation as well as DNA breaks caused by direct energy transfer.

Protective equipment available to the operator includes lead aprons, thyroid shields, lead eye protection, ceiling-suspended leaded shields, rolling leaded shields, radiation-attenuating sterile surgical gloves, and sterile lead-equivalent patient-mounted drapes.^40^ Below table lead shielding is particularly important for minimizing scatter radiation.^41^ The stark findings of the present study specifically highlight the importance of using leg-leaded pads by demonstrating that the markers of DNA damage detected in operators’ circulating lymphocytes were absent when the operator wore additional lower leg lead shielding. The electronic dosimeters placed over the leg confirmed a significant amount of scatter radiation absorbed at that level—in fact, higher than the dose at chest level. The capability of modern fixed-imaging systems to produce higher quality images compared with mobile c-arms is associated with a significantly higher amount of scatter radiation produced by the radiation source under the operating table.^42^ Operators often neglect to wear lower leg lead shielding, viewing its use as cumbersome and unnecessary, but the present data highlight the importance of protecting the legs.^43^

Dose awareness and training in radiation protection are fundamental for minimizing occupational radiation exposure. There is currently no mandatory requirement for training in fluoroscopic operation or mandatory radiation protection certification for vascular operators in the United Kingdom. However, radiation-exposed workers are encouraged to attend Ionising Radiation (Medical Exposure) Regulations training courses. Vascular surgery was granted specialty status in the United Kingdom in 2013, and with this a new curriculum and training program was developed. The first cohort of trainees has had instruction on radiation protection practices as part of their induction into the program. The first sitting of the examination for these trainees will take place in 2018 and should include questions on radiation safety, reflecting the content of the new curriculum.

The current endovascular case mix exposes the vascular operator to relatively high radiation doses compared with, for example, interventional cardiologists performing percutaneous coronary intervention. In our experience, the per case dose to the vascular operator is almost 6-fold higher during IEVAR compared with percutaneous coronary intervention, but it should be noted that interventional cardiologists perform cases more frequently (Table II in the online-only Data Supplement).

It is essential that operators adhere to “As Low as Reasonably Achievable” principles to minimize exposure to themselves, their team, and the patient. These principles include wearing a dosimeter at all times, with cumulative doses monitored regularly and leveraged use of real-time dosimetery where possible.^44,45^ Other principles include lowering the rate of fluoroscopy where applicable, minimizing the use of cinefluorography (which produces 10 times more radiation compared with standard fluoroscopy), using collimation, and maximizing the distance between the operator and x-ray source, remembering that as distance doubles exposure is reduced by a factor of 4.^46^ Finally, closely monitoring the orientation and angulation of the x-ray source, particularly avoiding left anterior oblique projection, which exposes the operating team to the highest amount of scatter radiation, is key for minimizing the absorbed dose.

### Limitations of This Study

The present study does not relate the effective radiation doses absorbed during each procedure, calculated by taking into account the radiosensitivity of different body tissues, to the expression of DNA damage/repair markers. We have also not established the relationship between the acute response (γ-H2AX and pATM levels) and cytogenetic markers of chronic low-dose radiation damage and DNA misrepair, such as micronuclei and dicentric chromosomes. Finally, it is important to stress that the biomarkers of radiation exposure measured in this study demonstrate an acute cellular response, but we do not yet know how this gives rise to an increased cancer risk. Relating one to the other would require longitudinal measurements in a much larger cohort of operators with long-term prospective follow-up.

## Acknowledgments

The authors acknowledge the members of the Guy’s and St Thomas’ Vascular Research Collaborative.

## Sources of Funding

Dr Modarai is funded by a British Heart Foundation Senior Clinical Research Fellowship (FS/17/24/32596).

## Disclosures

None.

## Supplementary Material

## Appendix

Guy’s and St Thomas’ Cardiovascular Research Collaborative, From Guy’s and St Thomas’ NHS Foundation Trust, London, United Kingdom: M Tyrrell, P Gkoutzios, S Abisi, S Black, H Zayed, RE Bell, M Sallam, L Biasi, SD Patel, T Donati, M Dialynas, B Sandford, S Redwood, S Perera, A Pavlidis, B Prendergast, J Gill.
