# Supplementary material for: Radiation-Induced DNA Damage in Operators Performing Endovascular Aortic Repair
Source: Circulation. 2017 Dec 18;136(25):2406–16. doi: 10.1161/CIRCULATIONAHA.117.029550 (PMC5753831; doi:10.1161/CIRCULATIONAHA.117.029550)
Supplement: Supplementary file 1 [file cir-136-2406-s001.pdf]

Gmail

Molly Klemarczyk <mklemarczyk@circulationjournal.org>

Re: CIRCULATIONAHA/2017/029550R1 Decision Letter

1 message

Patel, Ashish <ashish.s.patel@kcl.ac.uk> Mon, Oct 2, 2017 at 5:58 PM
To: Molly Klemarczyk <mklemarczyk@circulationjournal.org>, "Modarai, Bijan" <bijan.modarai@kcl.ac.uk>

Dear Molly,

I hope you are well.

I am writing to confirm that I give consent for my image to be published in Circulation (Fig 4B).

Please let me know if there is any other information that you need.

Best wishes,

Dr Ashish Patel

Ashish S Patel PhD MRCS
Clinical Lecturer in Vascular Surgery
Academic Department of Vascular Surgery
School of Cardiovascular Medicine and Science

From: Molly Klemarczyk <mklemarczyk@circulationjournal.org>
Sent: 02 October 2017 20:49:16
To: Modarai, Bijan
Cc: Patel, Ashish
Subject: Re: CIRCULATIONAHA/2017/029550R1 Decision Letter

Thank you, Bijan. I look forward to receiving an email from Dr. Patel giving his consent.

And yes, that is correct. Please confirm as soon as possible if you are going to change to an Open Access agreement. If so, please specify which Open Access License you would like to proceed with, and I will issue the necessary forms to all authors via email. Each author will need to fill out one form. As a reminder, you can compare licenses here: http://www.ahajournals.org/sites/default/files/additional-assets/Open%20Access/article_charges.pdf. Please let me know if you have any questions.

Many thanks,

Molly

On Sat, Sep 30, 2017 at 8:14 AM, Modarai, Bijan <bijan.modarai@kcl.ac.uk> wrote:

> This time cc'd!
>
> On 30 Sep 2017, at 13:14, Bijan Modarai <bijan.modarai@kcl.ac.uk> wrote:
>
> Thank you Molly
> I have cc'd Dr Patel so he can provide the statement required.
> You mentioned in a previous email that you would need all the authors to sign up for open access before you proceed? Or is this not required?
> Best
> Bijan
>
> On 29 Sep 2017, at 18:47, Molly Klemarczyk <mklemarczyk@circulationjournal.org> wrote:
>
> Hi Bijan,
>
> Excellent, thanks for letting us know. Actually all we'll need is a signed statement from Dr. Patel giving his consent, and we'll be all set to proceed.
>
> All best,
>
> Molly

On Thu, Sep 28, 2017 at 4:28 PM, Modarai, Bijan <bijan.modarai@kcl.ac.uk> wrote:

Dear Molly
The subject is Ashish Patel, one of the first authors. He would be happy to sign the release
Best
Bijan

On 28 Sep 2017, at 19:00, Molly Klemarczyk <mklemarczyk@circulationjournal.org> wrote:

Hi Bijan,

I also wanted to follow-up with you regarding the images. Thank you for confirming that 4B is original. Do I take this to mean you took this photograph? If so, we will need a release to be signed by the subject stating that they consent to have their photo published.

Many thanks,

Molly

On Wed, Sep 27, 2017 at 2:59 PM, Modarai, Bijan <bijan.modarai@kcl.ac.uk> wrote:

Thank you Molly
We will make a decision about open access as soon as possible. Do I understand correctly that the cheapest option for open access is $3200?
I am still not sure of the total cost as we don't yet know how many pages final article will be?

I can confirm that all images used are original including 4B

Best wishes
Bijan

Bijan Modarai PhD FRCS
Reader/Consultant in Vascular Surgery
Lead, Complex Endovascular Aortic Intervention

Academic Department of Vascular Surgery
St Thomas' Hospital | Cardiovascular Division
1st Floor North Wing, London, SE1 7EH | UK
PA (Mr Jason Alvey): +44 (0) 2071880216

On 26 Sep 2017, at 14:25, Modarai, Bijan <bijan.modarai@kcl.ac.uk> wrote:

Dear Molly
Thank you very much for your reply.

We have now uploaded the final files. I have used the cover letter that was uploaded with the resubmission - please let me know if you want this changed.

Just to confirm, I would like the following as the headline authors on Pubmed: Tamer El-Sayed, Ashish S Patel, Jun S Cho, James A Kelly, Francesca E Ludwinski, Prakash Saha, Oliver T Lyons, Alberto Smith, Bijan Modarai.
The rest as we discussed linked to pubmed search but appear as Guy's and St Thomas' Research Collaborative.

I would be grateful if you could give me an indication of when the paper will appear online and so I can put you in touch with any relevant media people.

I have included my twitter handle. I gather there is also an opportunity to do a Podcast? If so can you advise me how I do that?

Please could you also give me an indication of cost if we were to publish the relevant pages in colour. Also I am keen for the paper to be open access. How much would this cost please?

Bijan Modarai PhD FRCS
Reader/Consultant in Vascular Surgery
Lead, Complex Endovascular Aortic Intervention
British Heart Foundation Intermediate Fellow

Academic Department of Vascular Surgery
St Thomas' Hospital, Cardiovascular Division
1st Floor North Wing, London, SE1 7EH
PA (Mr Jason Alvey): +44 (0) 2071880216

On 20 Sep 2017, at 17:50, Molly Klemarczyk <mklemarczyk@circulationjournal.org> wrote:

Dear Dr. Modarai,

Thank you for your email; I would be happy to assist with your questions:

1. Yes, we would be pleased to index these individuals as collaborators in PubMed. In order to do so, please include a list of their names and affiliations at the end of the final manuscript under the heading "Appendix".

2. While neither the print publication or online, publish-ahead-of-print date have been determined quite yet, we should be able to determine the online release date around the end of next week. Once we have that settled, I would be happy to answer any questions from interested media parties about when they can release their own news. In the meantime, I would suggest that you carefully review our embargo policy on our website here: http://circ.ahajournals.org/content/journal-policies.

If you have any further questions, please let me know.

Kindest thanks,

Molly Klemarczyk

--

Molly Klemarczyk
Assistant Managing Editor | Circulation
American Heart Association
mklemarczyk@circulationjournal.org
1-781-902-4410

On Mon, Sep 18, 2017 at 9:37 AM, Circulation Journal <circ@circulationjournal.org> wrote:

> ---------- Forwarded message ----------
> From: **Modarai, Bijan** <bijan.modarai@kcl.ac.uk>
> Date: Sat, Sep 16, 2017 at 5:56 AM
> Subject: Re: CIRCULATIONAHA/2017/029550R1 Decision Letter
> To: "circ@circulationjournal.org" <circ@circulationjournal.org>
>
> Dear Dr Brilakis and Editorial team
> We are delighted with the decision on our manuscript.
> I had two questions if I may.
> The individuals acknowledged in the manuscript are part of our research collaborative and have had a significant input into the present study. Would it be possible to include them as associated (not headline authors) and have their names linked to PubMed?
> For example,
> **Guy's and St Thomas' Cardiovascular Research Collaborative:**
> Tyrrell M, Gkoutzios P, Abisi S, Black S, Zayed H, Bell RE, Sallam M, Biasi L, Patel SD, Donati T, Dialynas M, Sandford B, Redwood S, Perera S, Pavlidis A, Prendergast B and Gill J.
>
> On a separate note, we should have the final files back to you in the next few days. When do you envisage the publication date to be? UK based Cardiovascular media outlets may be interested in reporting the fact that this study is to be published by Circulation. What are the embargo date/other considerations for reporting the fact that it is to be published in Circulation and what the content of the study is?
>
> Thank you in advance
> Best wishes
> Bijan
>
> Bijan Modarai PhD FRCS
> Reader/Consultant in Vascular Surgery
> Lead, Complex Endovascular Aortic Intervention
> British Heart Foundation Senior Fellow
>
> Academic Department of Vascular Surgery
> St Thomas' Hospital, Cardiovascular Division
> 1st Floor North Wing, London, SE1 7EH
> PA (Mr Jason Alvey): +44 (0) 2071880216
>
> On 15 Sep 2017, at 13:33, "circ@msubmit.net" <circ@msubmit.net> wrote:
>
> Circulation
>
> September 15, 2017

Mr. Bijan Modarai, PhD FRCS
King's College London
Academic Department of Surgery
St Thomas' Hospital
London SE1 7EH
United Kingdom

RE: CIRCULATIONAHA/2017/029550R1
Radiation Induced DNA Damage in Operators Performing Endovascular Aortic Repair

Dear Mr. Modarai,

Thank you for returning your revised manuscript promptly. We are pleased to inform you that it is now acceptable for publication in ***Circulation***.

Pending receipt of all items required for publication, we plan to publish this paper as soon as possible.

1. Title Page:
a. Running title: Please provide a running title for your manuscript. A running title is an abbreviated version of your complete title with no more than 50 characters including spaces.
b. Affiliations: Please use numbers, rather than letters, to indicate affiliations.
c. Please include the Twitter handle for you and/or for the first author, if you have one.

2. Abstract: Please list your keywords at the end of your abstract.

3. References: Please provide full publication information for references 27 and 34 such as website, article title, publishing organization, authors, date published, date accessed, URL, etc.

4. Tables and Figures:
a. Please ensure that each table and figure is cited sequentially within the text.
b. Please remove your figures from the end of the manuscript, as final image files will need to be submitted separately. Please leave the figure legends intact.
c. Tables should be typed into the end of the manuscript and included as editable text. They should not be inserted as an image, or uploaded as a separate file.
d. Please refer to supplemental elements throughout the text as "Supplemental Fig 1" etc.
e. Please be sure to label the supplemental table that is currently unlabeled in the supplemental methods.
f. Authors
should obtain written permission from the original publishers of any reprinted
or adapted figures or tables. Please confirm the permission status of your
figures and email any permissions documentation to circ@circulationjournal.org.

5. Final files for the text and figures are required. Word is the acceptable format for your manuscript text and tables. Acceptable figure formats are TIFF and EPS. If a figure is created using PowerPoint, we prefer that you create a PDF using the save as PDF menu option, and submit that PDF file. You will receive an email containing a link to upload your best and final files.

6. Online Supplemental Data: A combined PDF of your supplemental data must be provided. The first page of this PDF should include the heading, "SUPPLEMENTAL MATERIAL." Please note that this single PDF should include all of the supplemental material related to your manuscript as follows: Supplemental Methods, Supplemental Tables, Supplemental Figures and Figure Legends, and Supplemental References. Please upload this PDF as a "supplemental publication material" file. Lastly, please note that the Supplemental Material is published as is and not copy edited. Please ensure the file is carefully reviewed for any grammatical errors.

Please note that once the journal receives all items required for publication, it will proceed with publishing ahead of print the accepted version of the manuscript as it appears as a PDF. When submitting the final files of the manuscript and figures, please ensure you have made any essential changes or corrections to content, grammar, and formatting. Once published ahead of print, the journal will be unable to make any revisions to the manuscript until it is published in print.

During the copyediting phase, there may be some changes in phrasing, but there will be no alteration of scientific content. When you receive your galley proofs, please read, correct, and return them

immediately to avoid delay in publication. Please note that it is your responsibility to make yourself available to review the proofs whenever they arrive, and if you are not available, publication of your manuscript will be delayed.

Lastly, please note that you will be charged a publication fee based on the authorship agreement that has been selected for this manuscript. Additional information on publication costs can be found at http://www.ahajournals.org/site/openaccess. Please notify the Editorial Office if you wish to pursue an Open Access license.

As the Copyright Transfer Agreement has been selected, the journal's publication fees are as follows:

Fee per printed black and white page: $70;
Fee per printed color page: $723;

We are very pleased to publish your interesting work. Thank you very much for the opportunity to work with you.

Sincerely yours,

Emmanouil Brilakis, MD
Associate Editor

Joseph A. Hill, MD, PhD
Editor-in-Chief
Circulation

--

Molly Klemarczyk
Assistant Managing Editor | Circulation
American Heart Association
mklemarczyk@circulationjournal.org
1-781-902-4410

--

Molly Klemarczyk
Assistant Managing Editor | Circulation
American Heart Association
mklemarczyk@circulationjournal.org
1-781-902-4410

--

Molly Klemarczyk
Assistant Managing Editor | Circulation
American Heart Association
mklemarczyk@circulationjournal.org
1-781-902-4410
