# Supplementary material for: Radiation-Induced DNA Damage in Operators Performing Endovascular Aortic Repair
Source: Circulation. 2017 Dec 18;136(25):2406–16. doi: 10.1161/CIRCULATIONAHA.117.029550 (PMC5753831; doi:10.1161/CIRCULATIONAHA.117.029550)
Supplement: Supplementary file 2 [file cir-136-2406-s002.pdf]

# SUPPLEMENTAL MATERIAL

## Supplemental Methods

**Flow cytometric analysis of γ-H2AX, pATM and OGG1 expression in lymphocytes**

Venous blood samples were collected from operators in EDTA tubes (BD Biosciences, UK). Red blood cells were lysed using Pharmlyse (BD Biosciences, UK) for 10mins and then washed in 0.5% BSA/PBS for 5mins at 4°C. Cells were stained with FITC-conjugated mouse anti-human CD3 antibody (Miltenyi Biotec, UK), Viogreen-conjugated mouse anti-human CD4 antibody, APC/Cy7-conjugated mouse anti-human CD8 antibody, PE/Cy7-conjugated mouse anti-human CD45RO antibody (BD Biosciences, UK) and PerCP-conjugated mouse anti-human CCR7 antibody (BioLegend, UK). Cells were fixed (Inside Fix, Miltenyi Biotec, UK) for 10mins at room temperature followed by permeabilisation on ice (Permeabilisation Buffer A, Miltenyi Biotec, UK) and washed twice prior to staining for γ-H2AX and phosphorylated ATM (pATM) using anti-human APC- and PE-conjugated antibodies respectively (BD Biosciences and BioLegend, UK). T cell subsets were divided into T Helper ($CD3^+CD4^+$) and cytotoxic ($CD3^+CD8^+$) cells. These were further phenotyped into Naïve ($CD3^+CD4^+CD45RO^-$ and $CD3^+CD8^+CD45RO^-$), central memory ($CD3^+CD4^+CD45RO^+CCR7^+$ and $CD3^+CD8^+CD45RO^+CCR7^+$) and effector memory ($CD3^+CD4^+CD45RO^+CCR7^-$ and $CD3^+CD8^+CD45RO^+CCR7^-$) helper and cytotoxic T lymphocytes respectively for further analysis of γ-H2AX and pATM expression. Samples from operators performing IEVAR were also analysed for the expression of PerCP-conjugated mouse anti-human OGG1 (Novus Biologicals, UK) in addition to γ-H2AX and pATM expression as previously described.

**Appendix**

**Guy's and St Thomas' Cardiovascular Research Collaborative**

From Guy’s & St Thomas’ NHS Foundation Trust, London, UK.

Tyrrell M, Gkoutzios P, Abisi S, Black S, Zayed H, Bell RE, Sallam M, Biasi L, Patel SD, Donati T, Dialynas M, Sandford B, Redwood S, Perera S, Pavlidis A, Prendergast B, Gill J.

## Supplemental Tables

### Supplemental Table 1. Antibodies used for flow cytometry

| **Antibody** | **Source** | **Conjugate** |
|---|---|---|
| CD3 | Miltenyi Biotec | FITC |
| CD4 | BD Biosciences | Viogreen |
| CD8 | BD Biosciences | APC/Cy7 |
| CD45RO | BD Biosciences | PE/Cy7 |
| CCR7 | BioLegend | PerCP |
| CD14 | BD Biosciences | Vioblue |
| CD45 | BD Biosciences | Vioblue |
| pATM | BioLegend | PE |
| γ-H2AX | BD Biosciences | APC |
| OGG1 | Novus Biologicals | PerCP |

**Supplemental Table 2. Radiation exposure during PCI and IEVAR**

**A**

| Radiation Exposure | PCI | IEVAR | *P* value |
|---|---|---|---|
| DAP (mGy.cm$^2$) | 9940 (4440-170170) | 82786 (53611-144257) | 0.01 |
| Screening Time (mins) | 11.2 (5.21-28.57) | 24.78 (14.06-49.39) | 0.007 |
| Operator personal dosimetry (µSv) | | | |
| Under lead | 0 (0-4) | 0 (0-3) | |
| Over lead | 6 (2-11) | 11 (4-74) | |
| Leg | 16 (2-48) | 92 (43-203) | 0.003 |

**B**

| Operator | Age | Years Performing Interventions | | Interventional sessions/week | Interventional cases / session |
|---|---|---|---|---|---|
| | | As Trainee | As Consultant | | |
| 1 | 46 | 8 | 9 | 3 | 3 |
| 2 | 62 | 8 | 22 | 2 | 4 |
| 3 | 53 | 5 | 18 | 4 | 4 |
| 4 | 36 | 6 | 1 | 3 | 5 |
| 5 | 52 | 5 | 16 | 2 | 3 |
| 6 | 42 | 8 | 5 | 1 | 3 |

**A**, Direct and indirect measurement of radiation exposure during PCI and IEVAR using DAP, fluoroscopy time and personal dosimetry (n=6). **B**, Details of age and caseload for interventional cardiologists that were performing the monitored procedures. PCI indicates percutaneous coronary intervention; IEVAR, infrarenal endovascular aortic repair; DAP, dose area product (mGy.cm$^2$).

# Supplemental Figures

**Supplemental Figure 1**

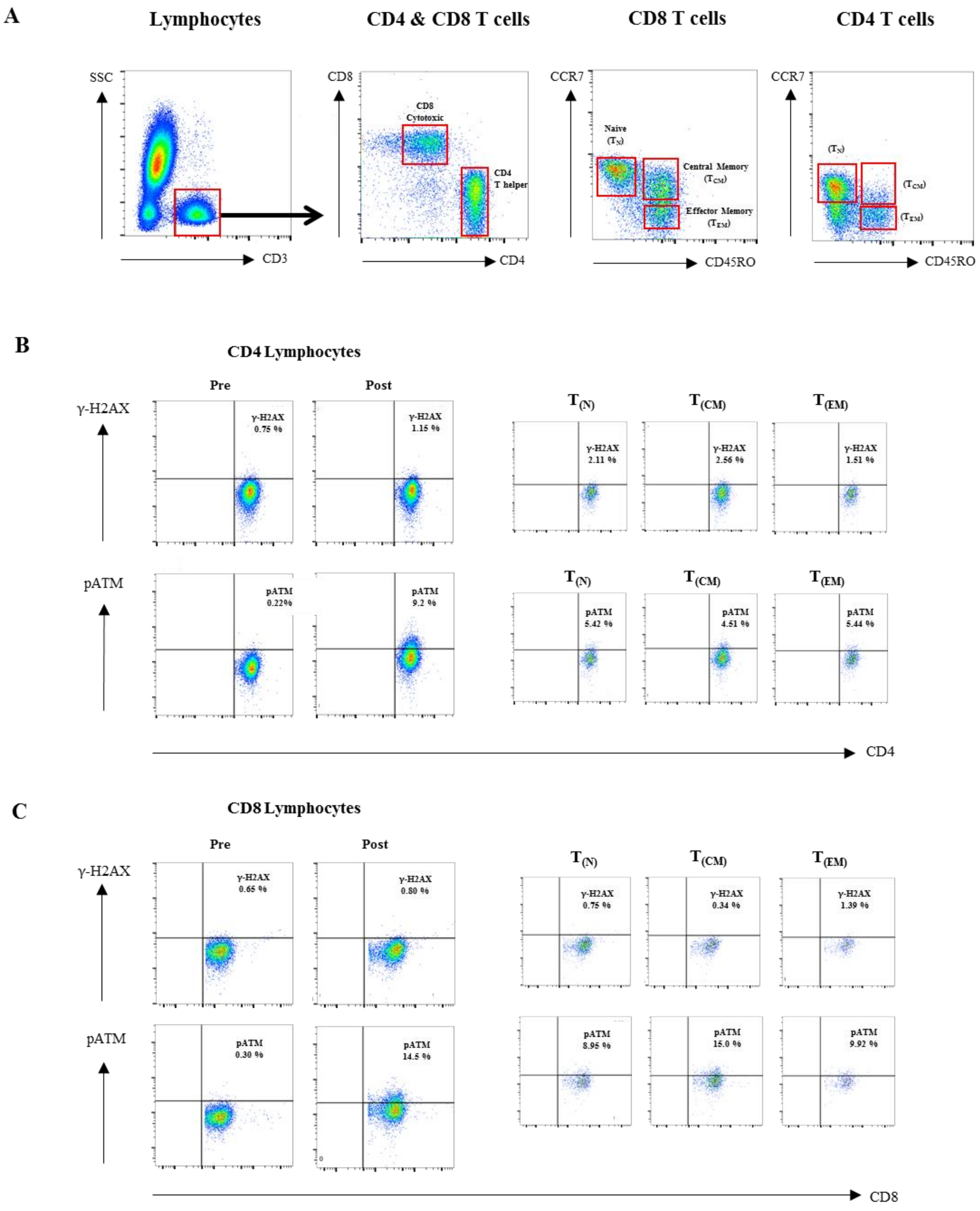

**Supplemental Figure 1. Flow cytometric analysis of γ-H2AX and pATM expression in subpopulations of T lymphocytes in operators during the peri-operative period of FEVAR**

**A,** Representative flow cytometric dot plots showing $CD3^+$ lymphocytes subdivided into $CD8^+$ (cytotoxic) and $CD4^+$ (T helper) cells. These two cell subsets are further phenotyped into $CD45RO^-CCR7^+$ (Naïve), $CD45RO^+CCR7^+$ (Central Memory, $T_{CM}$) and $CD45RO^+CCR7^-$ (Effector Memory, $T_{EM}$) cells. **B**, Example flow cytometric dot plots show increased γ-H2AX and pATM expression in $CD3^+CD4^+$ T helper cells and their naïve, $T_{CM}$ and $T_{EM}$ subpopulations from the blood of an operator immediately after FEVAR. **C**, Example flow cytometric dot plots show an increase in γ-H2AX and pATM expression in $CD3^+CD8^+$ cytotoxic T cells and their naïve, $T_{CM}$ and $T_{EM}$ subpopulations from the blood of an operator immediately after FEVAR. γ-H2AX indicates gamma H2AX; pATM, phosphorylated ataxia telangiectasia mutated protein; FEVAR, fenestrated endovascular aortic repair.

**Supplemental Figure 2**

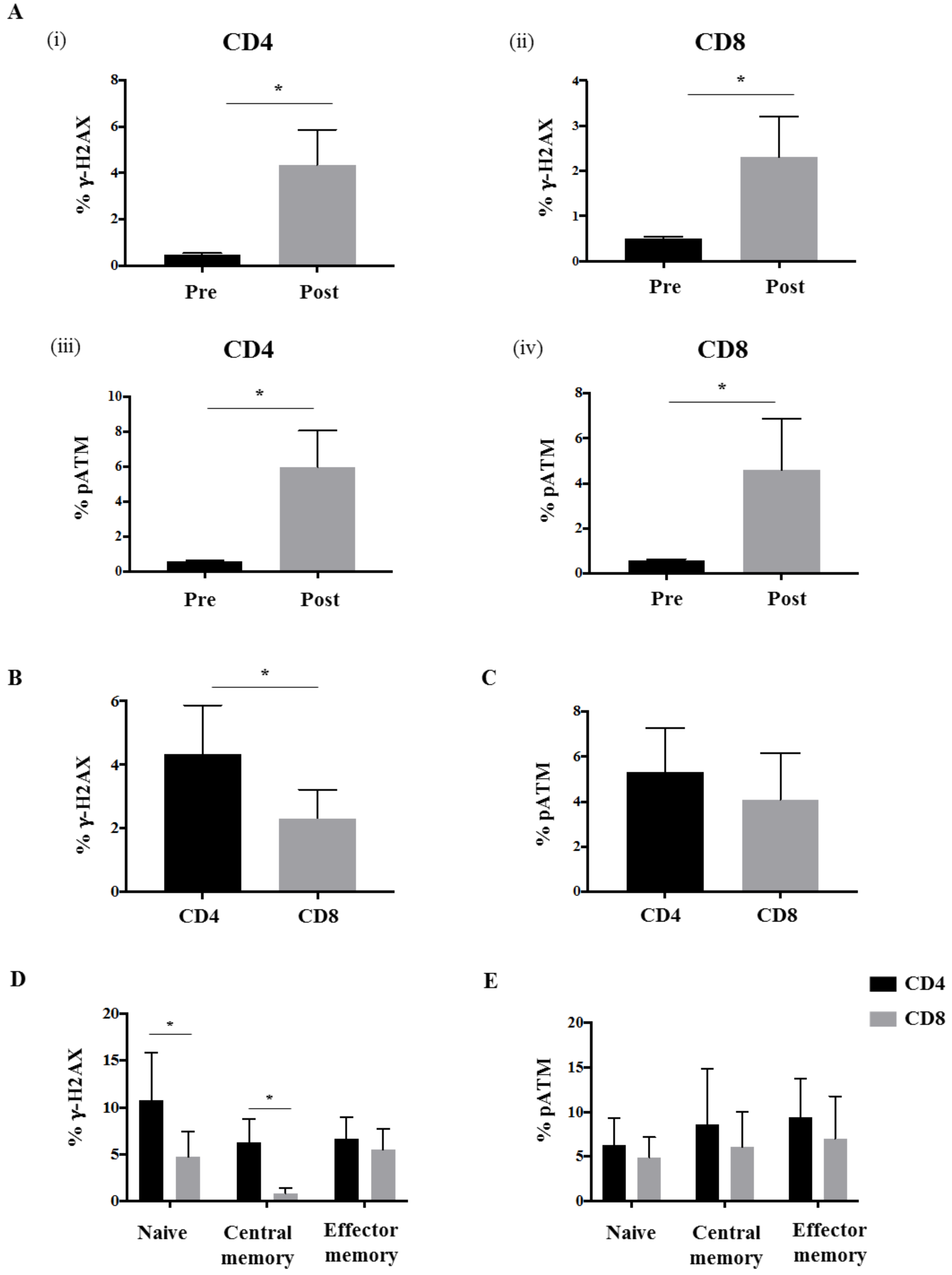

**Supplemental Figure 2. Comparison of changes in γ-H2AX and pATM in T helper and cytotoxic T cell subsets during the peri-operative period of FEVAR**

**A,** The post-operative expression of γ-H2AX was significantly higher in (i) $CD4^+$ T helper cells (n=6, $P<0.02$) and (ii) $CD8^+$ T cytotoxic cells (n=6, $P<0.02$) of operators performing BEVAR/FEVAR. The expression of pATM was similarly raised in (iii) $CD4^+$ T helper (n=6, $P<0.04$) and (iv) $CD8^+$ T cytotoxic cells (n=6, $P<0.04$). **B,** The post-operative γ-H2AX expression was higher in $CD4^+$ T helper cells compared with $CD8^+$ cytotoxic T cells in operators after FEVAR (n=6, $P<0.04$). **C,** The post-operative expression of pATM in $CD4^+$ T helper cells and $CD8^+$ cytotoxic T cells was comparable (n=6). **D,** The post-operative expression of γ-H2AX was significantly higher in $CD4^+$ Naïve and Central memory cells compared with their $CD8^+$ counterparts (n=6, $P<0.05$). **E,** The post-operative expression of pATM in Naïve, Central Memory and Effector memory cells was comparable in $CD4^+$ T helper cells and $CD8^+$ cytotoxic T cells. *$P<0.05$. γ-H2AX indicates gamma H2AX; pATM, phosphorylated ataxia telangiectasia mutated protein; FEVAR, fenestrated endovascular aortic repair.

**Supplemental Figure 3**

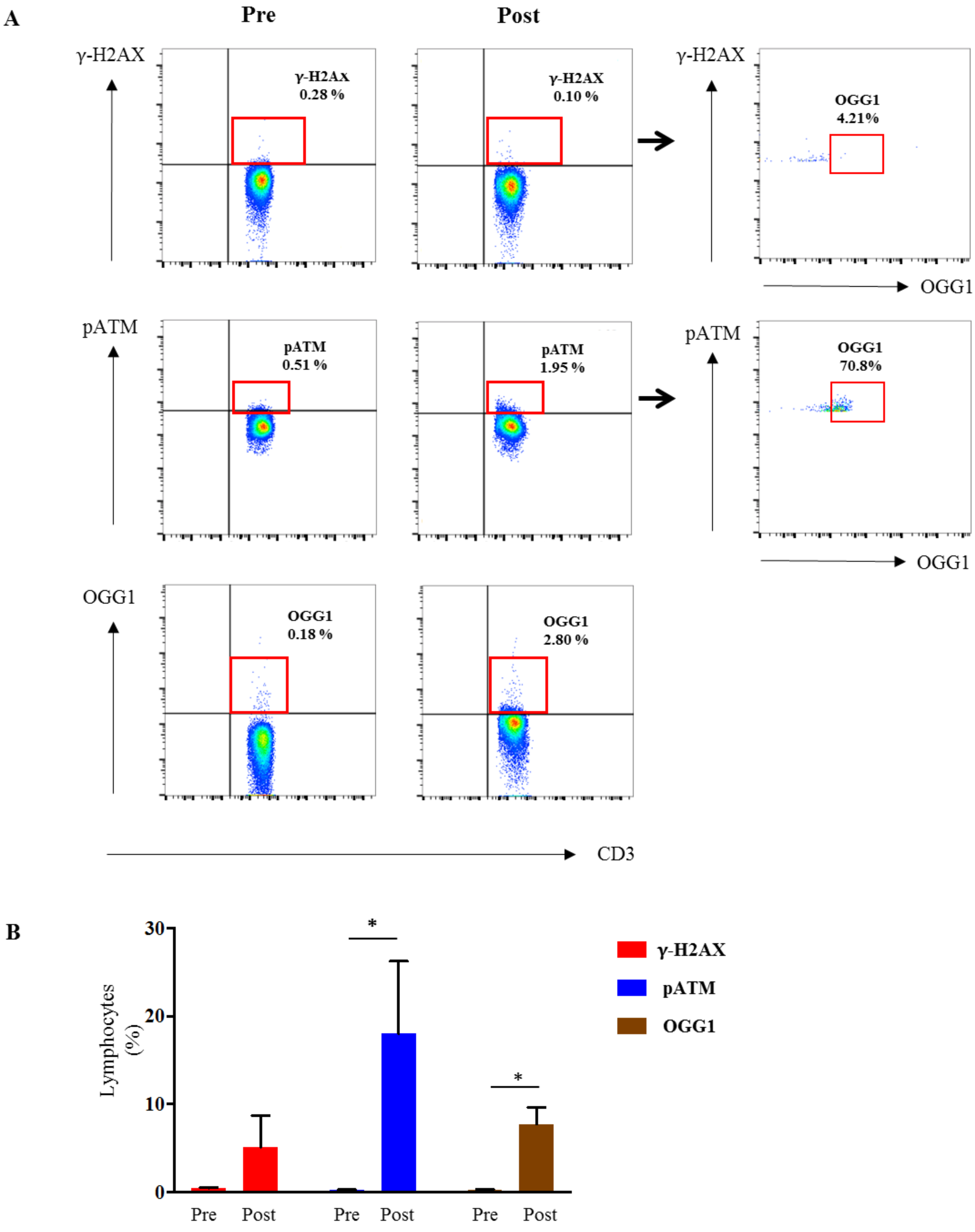

**Supplemental Figure 3. Expression of OGG1 on γ-H2AX and pATM expressing CD3+ T lymphocytes during the peri-operative period of IEVAR**

**A,** Flow cytometric analysis of blood taken from an operator before and after IEVAR showing an increase in lymphocyte expression of pATM and OGG1, but not γ-H2AX in CD3+ lymphocytes. Selective gating of γ-H2AX and pATM positive cells indicates a greater increase in OGG1 expression in the pATM positive population. **B,** Analysis of CD3+ lymphocytes, isolated from 6 operators after IEVAR, co-stained for γ-H2AX, pATM and OGG1 shows a significant increase in pATM and OGG1 ($P$<0.03, both), but not γ-H2AX. IEVAR indicates infrarenal endovascular aortic repair; γ-H2AX, gamma H2AX; OGG1, 8-oxoguanine glycosylase; pATM, phosphorylated ataxia telangiectasia mutated protein.

**Supplemental Figure 4**

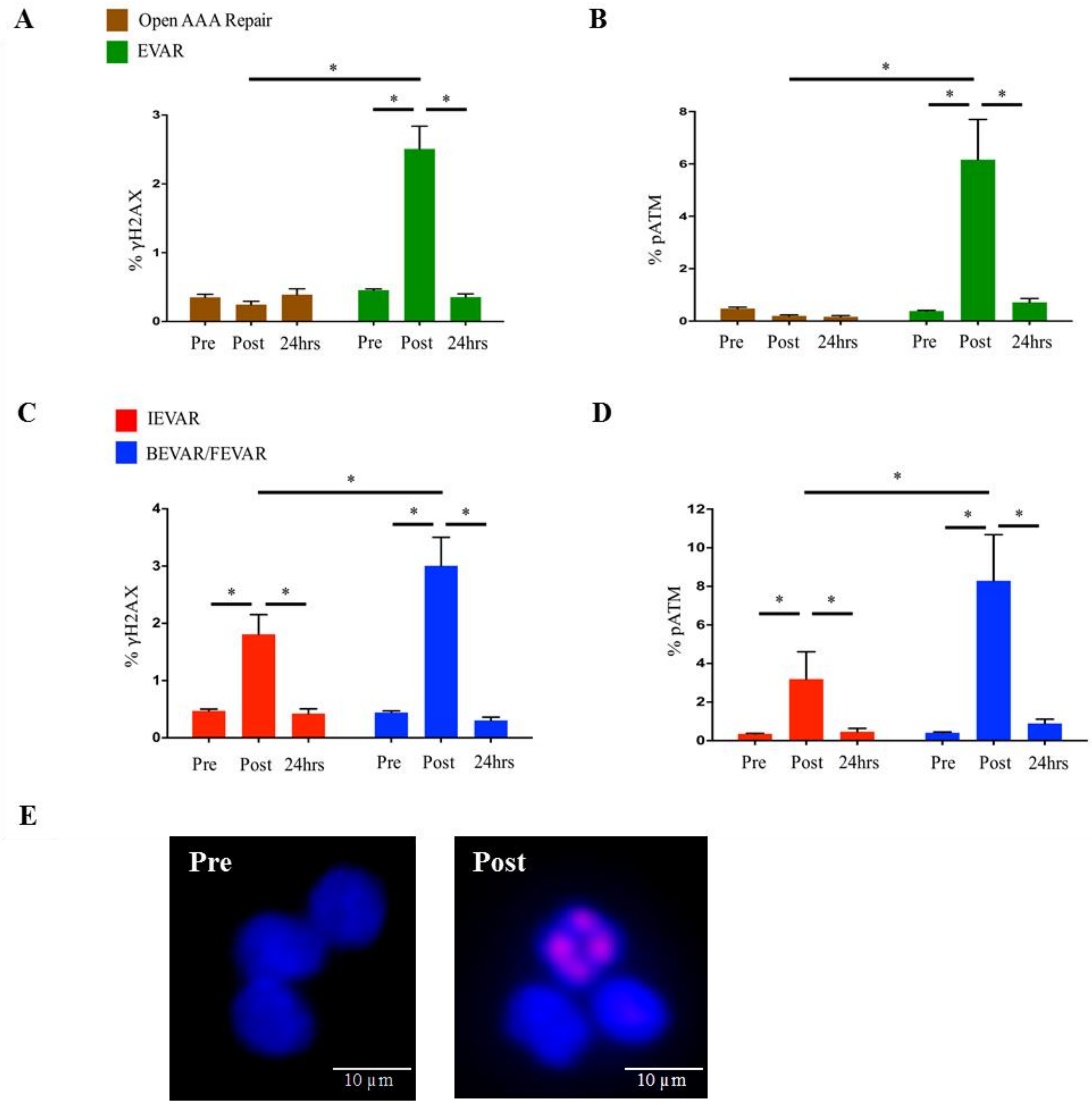


**Supplemental Figure 4. Patient response to radiation exposure during EVAR**

**A,** Expression of γ-H2AX in lymphocytes prior to, immediately and 24 hours after open aortic repair (n=14) and EVAR (all IEVAR and BEVAR/FEVAR procedures grouped together, n=72). **B,** Expression of pATM in lymphocytes prior to, immediately and 24 hours after open aortic repair (n=14) and EVAR (n=72). **C,** γ-H2AX expression during the peri-

operative period of BEVAR/FEVAR (n=42) compared with IEVAR (n=30). **D,** pATM expression during the peri-operative period of BEVAR/FEVAR (n=42) compared with IEVAR (n=30). **E**, Immunohistochemical staining of lymphocytes (DAPI, blue) isolated from a patient shows, compared with the pre-operative sample, an increase in γ-H2AX expression (purple foci) in these cells immediately after EVAR. *$P<0.05$. γ-H2AX indicates gamma H2AX; pATM, EVAR, endovascular aortic repair; IEVAR, infra-renal endovascular aortic repair; BEVAR, branched endovascular aortic repair; FEVAR, fenestrated endovascular aortic repair; phosphorylated ataxia telangiectasia mutated protein. DAPI, 4',6-Diamidino-2-Phenylindole Dihydrochloride, scale bar =10μm.
